# Extraction of High-Value Chemicals from Plants for Technical and Medical Applications

**DOI:** 10.3390/ijms231810334

**Published:** 2022-09-07

**Authors:** Pritam Kapadia, Amy S. Newell, John Cunningham, Michael R. Roberts, John G. Hardy

**Affiliations:** 1 Department of Chemistry, Lancaster University, Lancaster LA1 4YB, UK; 2 Lancaster Environment Centre, Lancaster University, Lancaster LA1 4YQ, UK; 3 Centre for Global Eco-Innovation, Lancaster University, Lancaster LA1 4YQ, UK; 4 CO_2_ Extraction Ltd., 7 Stevant Way, Northgate, Morecambe LA3 3PU, UK; 5 Materials Science Institute, Lancaster University, Lancaster LA1 4YB, UK

**Keywords:** biomolecules, essential oils, secondary metabolites, high-value chemicals, extraction methods, applications

## Abstract

Plants produce a variety of high-value chemicals (e.g., secondary metabolites) which have a plethora of biological activities, which may be utilised in many facets of industry (e.g., agrisciences, cosmetics, drugs, neutraceuticals, household products, etc.). Exposure to various different environments, as well as their treatment (e.g., exposure to chemicals), can influence the chemical makeup of these plants and, in turn, which chemicals will be prevalent within them. Essential oils (EOs) usually have complex compositions (>300 organic compounds, e.g., alkaloids, flavonoids, phenolic acids, saponins and terpenes) and are obtained from botanically defined plant raw materials by dry/steam distillation or a suitable mechanical process (without heating). In certain cases, an antioxidant may be added to the EO (EOs are produced by more than 17,500 species of plants, but only ca. 250 EOs are commercially available). The interesting bioactivity of the chemicals produced by plants renders them high in value, motivating investment in their production, extraction and analysis. Traditional methods for effectively extracting plant-derived biomolecules include cold pressing and hydro/steam distillation; newer methods include solvent/Soxhlet extractions and sustainable processes that reduce waste, decrease processing times and deliver competitive yields, examples of which include microwave-assisted extraction (MAE), ultrasound-assisted extraction (UAE), subcritical water extraction (SWE) and supercritical CO_2_ extraction (scCO_2_). Once extracted, analytical techniques such as chromatography and mass spectrometry may be used to analyse the contents of the high-value extracts within a given feedstock. The bioactive components, which can be used in a variety of formulations and products (e.g., displaying anti-aging, antibacterial, anticancer, anti-depressive, antifungal, anti-inflammatory, antioxidant, antiparasitic, antiviral and anti-stress properties), are biorenewable high-value chemicals.

## 1. Introduction

The strive for sustainability in industry motivates the consideration of plants as a source of biorenewable feedstocks, due to their abundance, the range of molecules they produce and our ability to employ engineering biology to generate new/valuable biomolecules [1,2,3]. Plants produce primary metabolites, which are common to most organisms (such as fats, proteins and sugars), and the more diverse secondary metabolites (species-specific, expressing various bioactives [4,5], and potentially therapeutic [6], used for the treatment of cancer, heart disease, circulatory disease and viral infection [7]).

**Figure 1:**
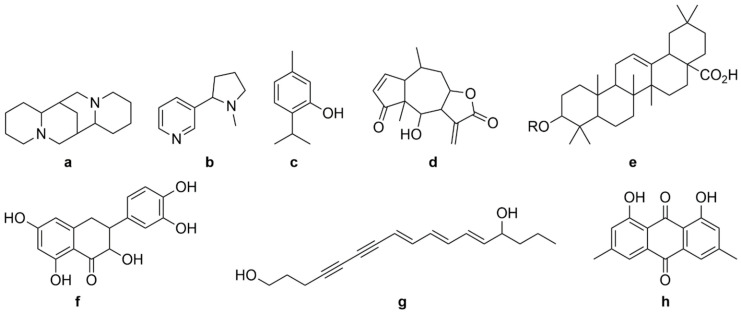
General structures of different categories of plant bioactive compounds: alkaloids (**a**,**b**), monoterpenes (**c**), sesqueterpenes (**d**), triterpenes, saponins, steroids (**e**), flavonoids (**f**), polyacetylenes (**g**), polyketides (**h**).

Biomolecules extracted from plant material may display bioactivity [8] (e.g., as natural preservatives for food [9], perfumes [10], etc.), resulting in their incorporation in real-world products. Natural variation in the molecular makeup of plant biomass has necessitated studies seeking to understand the factors influencing variations in the molecular composition/yield after employing a specific extraction method (species, location/environment, harvest time, plant maturity, genetic and physiological factors [10,11,12,13,14]) to facilitate the harvest of a rich and diverse portfolio of molecules [11]. The majority of these molecules may be classified into several sub-groups of chemical species, including alkaloids, monoterpenes, sesqueterpenes, triterpenes, saponins, steroids, flavonoids, polyacetylenes and polyketides. Some representative structures are illustrated in Figure 1.

Terpenes (terpenoids) are constituents of plant biomass and are known to be responsible for many of the medicinal and pharmacological applications of chemicals extracted from plants [15,16], resulting from their antimicrobial, antibacterial, anticancer, anti-malaria and anti-inflammatory properties [11,17]. Alkaloids, flavonoids, phenolic acids and saponins are also observed to have pharmacological benefits, including anti-inflammatory, antioxidant, anti-microbial, anti-diabetic, anti-mutagenic, anti-spasmodic, hepato-protective and immune-stimulant properties [18,19]. A huge variety of biomolecules have been reported, including >25,000 terpenes, >12,000 alkaloids and >8,000 phenolic compounds, the structures of which govern their physicochemical properties and pharmacological behaviour [4]. The typical characteristic of terpene molecules is the presence of a five-carbon isoprene (2-methyl-1,3-butadiene) base unit, which may be considered the building block of terpenes [20]. Several permutations of terpenes exist, depending on the number of isoprene units they contain. Monoterpenes contain two isoprenes; sesquiterpenes contain three isoprenes; diterpenoids contain four isoprenes; sesterpenes contain five isoprenes; triterpenes contain six isoprenes; and meroterpenes are a broad class of compounds with partial terpenoid skeletons [21]. Alkaloids are identified by the presence of one or more nitrogen atoms as amines/amides/heterocycles [22]. Flavonoids are polyphenolic compounds containing benzo-gamma-pyrone structures [23], wherein the number and position of hydroxyl groups influences the antioxidant potential of the compound [24]. Saponins are glycosides containing sugar chains attached to triterpenes or sapogenins, rendering them amphiphilic and enabling their utilisation in detergents and wetting, emulsifying and foaming agents [25].

Secondary metabolites are not of direct use for a plant’s growth/reproduction but play important roles in an organism’s interaction with its environment, functioning as chemical defences that mitigate the impacts of biotic and abiotic stresses [26,27]. This is fundamental for understanding the nature of these compounds and their commercialisation, and this field of study (allelopathic activity) is defined as “The science that studies any process involving secondary metabolites produced by plants, algae, bacteria and fungi that influences the growth and development of agricultural and biological systems” [11]. These molecules, known as allelochemicals, may have both detrimental and beneficial effects on a target organism [28]. It is important to consider this, as the default effects of these secondary metabolites may have differing results when introduced into an unintended organism. For instance, caffeine, a purine alkaloid, is originally synthesised by plants for its antimicrobial activity and as a natural insecticide. However, when ingested by people, it results in both analgesic and stimulatory responses by improving alertness, vigilance, attention, reaction time and attention [29,30], which are in direct contrast to its natural use. Likewise, untested and unidentified compounds [31] within a plant feedstock may also have unpredictable effects on users.

Plants produce aromatic compounds that may have attractive scents designed to attract pollinators and facilitate seed dispersion [31], which can be extracted from specimens, including flowers, barks, roots, fruits, leaves and other parts of plants. These include many volatile chemicals, which are often unsaturated hydrocarbons, alcohol, aldehydes, esters, ethers, ketones, phenols and terpenes [32]. The diverse nature of these compounds, coupled with interspecies and intraspecies variation, results in a large portfolio of possible applications of compounds extracted from plants (e.g., see Table 1). The full economic, health and societal benefits of these chemicals necessitates efficient extraction methods and the analysis of the constituent compounds. Conventional methods employed for the extraction of biomolecules typically utilise distillation for volatile components or cold pressing. Greener alternatives have been developed (extraction methods employing solvents of various types, including organic/aqueous solvent systems, microwaves, ultrasound and supercritical CO_2_, as outlined below) [33,34], that subsequently employ analytical techniques, such as gas chromatography (GC) or high-performance liquid chromatography (HPLC) coupled with an appropriate mass spectrometry (MS) system (i.e., GC-MS and HPLC-MS), in order to identify the compounds isolated.

Molecules extracted from plant material are industrially interesting because of their therapeutic, medicinal and biochemical properties [35,36,37], resulting in their potential applications as antiseptics, anti-inflammatories, cosmetics, flavourings, fragrances, preservatives and sedatives [8], enabling the treatment of numerous diseases and ailments [8,38,39], which explains their popularity in healthcare formulations [31]. The planet’s rich biodiversity means that bioactive molecules may already be identified or, indeed, as yet unidentified.

A promising mode of application of the biomolecules produced by plants is the fight against pathogens displaying antimicrobial resistance, such as alkaloids, cyanogenic glucosides, phenolics, terpenoids, steroids, etc., which have a bioactivity that results in degradative physiological changes to the pathogens [40]. The fact that many plant extracts are relatively unexplored/under-investigated highlights their potential for other high-value applications (e.g., as antimicrobials/antivirals [41]). While herpes simplex virus is typically treated with acyclovir and other synthetic drugs, issues related to their efficacy and side effects have led to the successful exploration of plant-derived biomolecules as a means of combating such infections [42].

The utilisation of plant-derived biomolecules to combat diseases such as cancer is motivated by a desire to minimise treatments including surgery, radiotherapy, chemotherapy and immunotherapy, and their concomitant side effects on patients [43]. It is also important to note that, although plant-derived biomolecules create an opportunity for their use in cancer therapy, some biomolecules (e.g., some of those extracted from *Salvia sclarea* and *Melaleuca quinquenerviaxi*) have been shown to induce oestrogen-dependent cancers. Indeed, certain molecules present in plants, such as cyanine, flavins, porphyrins and psoralen, may be carcinogenic [8]. Plant-derived biomolecules may be used for their anticancer effects, which are derived from their ability to regulate the production of reactive oxygen species (ROS), which are associated with inflammation, oxidative stresses and signalling pathways that may lead to cancer and tumour development. Alternatively, plant-derived biomolecules may also be utilised for inducing apoptosis as a means of coping with cancer [8,31,44]. Plant-derived biomolecules may also find application in methods for coping with the indirect factors of diseases such cancer on a patient’s health, such as anxiety and insomnia, and their use has been shown to be effective for treating both of these [45], as exemplified by lavender-derived biomolecules, which have been shown to improve sleep quality and reduce anxiety [46,47].

Plant-derived biomolecules may be utilised for the treatment of numerous other mental ailments, further to their use in anxiety and insomnia. Their use has potential for the treatment of Alzheimer’s disease (AD), a neurodegenerative disease associated with memory and cognition [48]. A major therapeutic strategy for AD is the inhibition of the enzyme acetylcholinesterase. Aromatherapy (exploiting the volatile components of plants) also provides an effective non-pharmacological therapy that can be used against neurodegenerative diseases; for instance, volatiles from rosemary/lemon and lavender/orange proved effective for addressing symptoms and cognition [48,49]. Nervonic acid, a chemical constituent of plant seeds, enhances brain health through biosynthesis and the maintenance of nerve cell myelin. It therefore aids in the repair of nerve pathways, providing an effective treatment against several mental ailments, including schizophrenia, psychosis and alcoholism [50]. The *Acer* genus has been identified as a promising raw material used to produce nervonic acid [50], but several other species have also been identified, which may prove to be useful resources in various environments, including, but not limited to, *Lunaria annua* seed oils, which have ~25% nervonic acid, and *Cardamine graeca*, which have ~45% [51]. Other notable species include *Tropaeolum speciosum*, *Borago officinalis* and *Cannabis sativa*, all of which contain nervonic acid in their seed oils and may therefore contribute to the treatment of neurological disorders and, in general, pharmacological and nutraceutical applications resulting from their bio-functionality [52].

Plant-derived biomolecules have also shown promising signs in the area of dental care. Commercial brands such as LISTERINE^®^ have incorporated them into mouthwashes for domestic use, which offers strong evidence to support the notion that plant-derived biomolecules have anti-plaque and anti-gingivitis properties [53,54]. It is also in competition with chlorhexidine, first investigated over 50 years ago, and is one of the most widely used oral antiseptics. Although both may stain teeth, chlorhexidine may also stain the tongue and gingiva and result in bitter and salty taste reductions [53]. A potential benefit may then be argued for the use of plant-derived biomolecules as a better alternative. Plant-derived biomolecules integrated into herbal toothpastes may exhibit antibacterial properties, which aid in tackling *Streptococcus mutans*, a bacterium known to result in the initial formation of tooth decay [55]. This activity may be the result of phytochemical biomolecules, such as menthol and eugenol, which influence the overall biological activity of their admixtures [56]. A study also concluded that the use of mouthwash containing plant-derived biomolecules alongside curcumin aided in supplementary treatments for reducing rheumatoid arthritis and chronic periodontitis activity [57], suggesting that plant-derived biomolecules, within oral/dental care, may be used as supplementary therapeutic agents for fighting against other diseases, in addition to their biological activities, which act as agents in dental care.

As early as 4500 BCE, in ancient Egypt there, was documentary evidence of the use of plant-derived biomolecules in cosmetics and ointments [31], and between 3000 to 2000 BCE, there were reports of the use of plants and their constituents for medicinal purposes, as well as fragrances in ancient India [37,58]. While various methods have been used to extract plant-derived biomolecules, only a handful are competitive on the industrial scale today.

Hydro-distillation (HD) involves the boiling of an aqueous suspension of plant material within an alembic, followed by the distillation of the resulting steam and plant-derived vapor, the collection of the condensate and the separation of the volatiles from the aqueous phase [59,60]. The closely related steam distillation utilises steam to volatilise plant-derived biomolecules that are of interest [34,59,60]. Solvent extractions involve the suspension of plant materials within organic solvents, followed by heating, filtration and solvent evaporation to isolate a filtrate, commonly in the form of a resin (resinoid) or a mixture of wax, fragrance and oils [59,60]. Cold pressing involves the mechanical compression of plant biomass to release components in the form of a watery emulsion, which is centrifuged to separate any hydrophobic oils [60,61]. Soxhlet extractions are a popular method, using a solid–liquid contact medium to remove compounds from a solid/gel matrix through their dissolution into a refluxing liquid phase [34].

The desire for innovation has resulted in the development of methods with reduced energy demands and CO_2_ emissions [62]. Microwave-assisted extraction (MAE) uses a microwave oven to irradiate and promote heat generation within the plant material, so that the energy load, CO_2_ emissions, costs and overall process time are reduced [34,63,64,65]. The applicability of MAE is quite broad relative to other methods, giving rise to various permutations of MAE, including compressed air microwave distillation (CAMD), vacuum microwave hydro distillation (VMHD), microwave hydro distillation (MWHD), solvent-free microwave extraction (SFME) and microwave-accelerated steam distillation (MASD) [63,65]. Similar to MAE, ultrasound-assisted extraction (UAE) is another option. UAE uses high frequency pulses that are applied to the sample to induce the cavitation phenomenon, which subsequently increases the rate of the mass transfer of molecules into the solvent [66,67,68]. Subcritical water extraction (SWE) operates by raising the temperature of the water to between 100–374 °C and applying a pressure high enough to maintain a liquid state. As the temperature is raised, there is an increase in the diffusion rate, as well as a decrease in the surface tension and viscosity. This may be optimised for the solubility of polar molecules, which is higher at lower temperatures, and less polar molecules, which have a higher solubility at higher temperatures [69,70]. Supercritical CO_2_ (SC-CO_2_) extraction operates by raising the temperature and pressure of CO_2_ above its critical points, 31.2 °C and 7.38 MPa, in order to attain a supercritical state. SC-CO_2_ may then be used as a solvent for molecule extraction, and by varying the temperature and pressure, the selectivity of these molecules may also be optimised. SC-CO_2_ passes through the feedstock to load bioactive molecules of the plant. The resulting discharge is then decompressed, enabling the separation of extracts with no remaining solvent residue, as the CO_2_ is allowed to evaporate [59,60,71].

Each method comes with it its own series of pros and cons in terms of what material may be optimised for a specific method, the sensitivity to the extraction method, yield, purity, cost and toxicity, as well as the specificity to various compounds within the feedstock, as outlined below. However, owing to the breadth of the literature, it is impossible to offer a comprehensive guide to the advantages and disadvantages of the extraction techniques discussed herein, as these are clearly compound-specific. However, the avid reader is directed towards some interesting literature, mentioned in the specific sections below, and some excellent reviews [66,72,73,74,75,76].

## 2. Extraction Methods for High-Value Chemicals

### 2.1. Hydrodistillation

**Figure 2:**
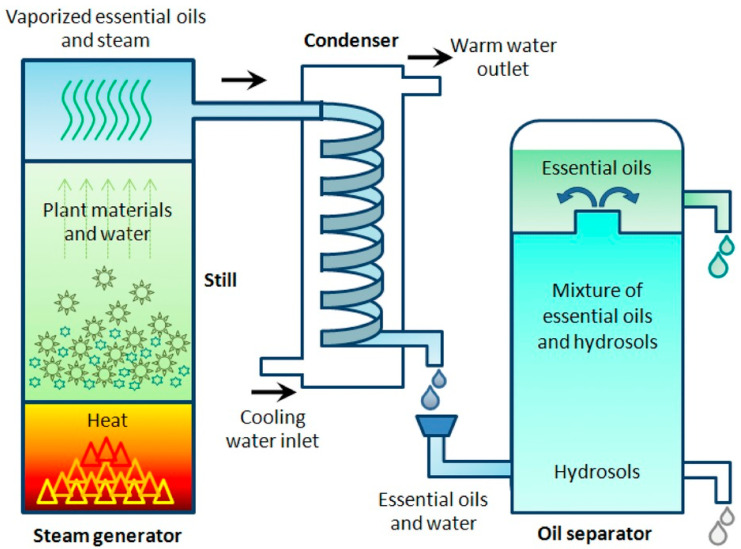
Diagrammatic illustration of the hydrodistillation method. Reproduced from [60] with permission.

Hydrodistillation is commonly applied in industry. However, due to the effects of temperature and pH within the distillation process, it has been found to result in potential alterations to the chemical composition of plant-derived biomolecules. This results from hydrolysis or the solubilisation of sensitive molecules [77,78]. However, typically, HD employs lower temperatures in order to reduce the risk of chemical decomposition. This is also advantageous, as it enables extraction from delicate flowers that would not survive typical distillation at temperatures above 100 °C. HD is also a method for easily extracting low-volatility and non-water-soluble molecules [30,59,60]. The outcomes of HD are feedstock specific, with HD typically being used for hydrophobic molecules with high boiling points within wood and plant material. The process is outlined in Figure 2 [59,60]. In some cases, HD extraction may damage and alter the chemical composition of the feedstock, but in others, the extract composition will be unaffected by the process conditions, enabling successful extraction.

Process times can vary depending on the feedstock and extraction specificity, with increasing distillation time resulting in increasing yields (process times typically vary between 5 and 240 min); however, this will be context-specific, based on the apparatus/feedstocks [79,80,81,82,83]. An important benefit of HD is the lack of toxic residues left on the products after extraction due to the use of water for the extraction process. HD also offers the benefit of its relatively inexpensive capital costs, resulting from the lack of steam required compared to steam distillation. The capital costs would include the construction of industrial stills, varying from 1000–2000 L capacities, which are typically made of copper, tinned on the inside and surrounded by insulating brick [84]. The large capacity enables the offset of the low yield provided by HD, equating to 1–2% by weight, in the case of fresh aromatic plants, with values as low as a 0.015% yield for the distillation of roses [85]. One key drawback of HD is the heat/energy requirements and subsequent CO_2_ emissions, which have led to innovative technologies aiming to reduce the energy consumption and costs and to increase the quality [86]. Whereas conventional methods can result in more than 70% of the total process energy being spent on the extraction process, methods such as MAHD seek to accelerate extraction while offering similar extraction compositions, with more effective heating, reductions in thermal gradients, higher yields and a more efficient process [87,88].

### 2.2. Steam Distillation

**Figure 3:**
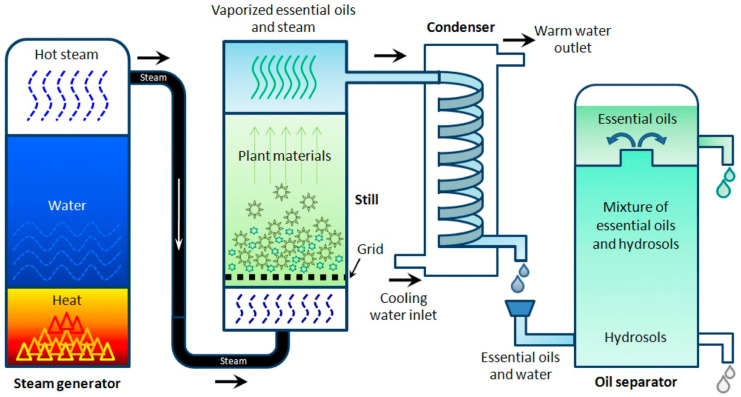
Diagrammatic illustration of the steam distillation method. Reproduced from [60] with permission.

Steam distillation is a method similar to that of hydro-distillation, but, in this case, the feedstock is treated directly with steam, which breaks down the cellular structures within the plant feedstocks, opens cavities containing the volatile components and enables them to volatilize for subsequent condensation and collection. The process is depicted in Figure 3 [60,89,90]. Steam distillation is also noted for its ability to be used for extractions of leaves and flowers [91], rendering it very popular in comparison with other methods of extraction [92]. The popularity of this method is indicative of its applicability to the distillation of polar, acidic and basic organic compounds of reasonable volatility [93]. However, polar compounds may be lost in the aqueous distillate and to the water within the still, which may require recovery through the redistillation of the water via cohobation, with the cost of added energy consumption. A subsequent consideration is the non-selective nature of steam distillation, leading to undesired extractions and the potential hydrolysis of active components resulting from elevated temperatures [94,95]. 

In summary, the advantages and disadvantages of steam distillation are as follows. The advantages include: solvent-free products, a lack of requirement for subsequent separation, a potential for industrial scale processing, inexpensive equipment and technological maturity. By comparison, the disadvantages include: the thermal degradation/hydrolysis of sensitive compounds, lengthy extraction times (1–5 h) and high energy consumption levels [95,96]. However, the extraction time requirements are context-dependent, with the multi-stage steam distillation extraction of volatiles from *Rosemarinuse officinialis* L. potentially being <1 h, although longer times have led to greater yields [97]. An investigation of the influence of steam distillation extraction times on the chemical composition of Patchouli oil concluded that increasing the extraction time only increases the quality of the resulting oil to an extent [98]. There is no simple rule regarding appropriate steam distillation extraction times due to variations in feedstocks, etc. [99].

### 2.3. Cold Pressing

**Figure 4:**
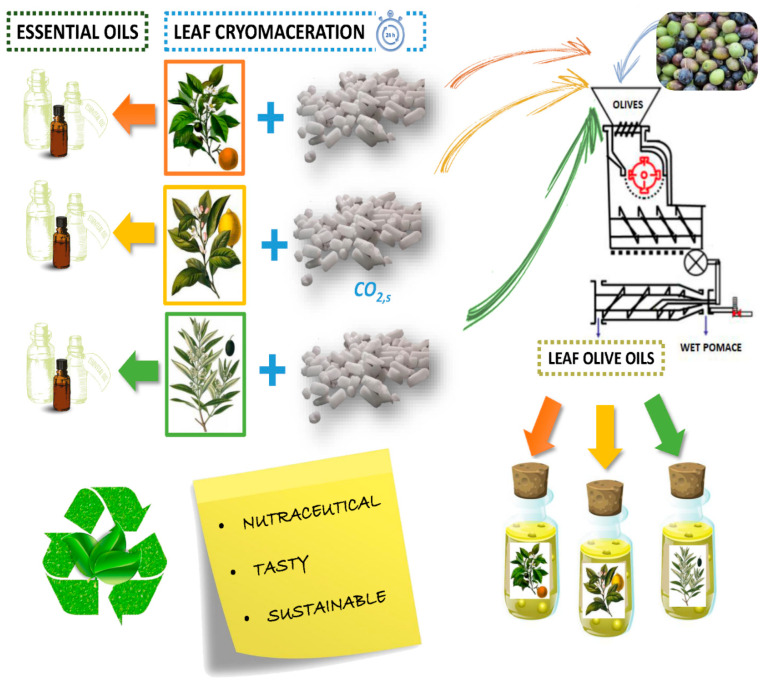
Schematic of the cold pressing setup. Reproduced from [109] with permission (open access, Attribution 4.0 International (CC BY 4.0)).

Cold pressing (illustrated in Figure 4) has been noted to be an effective method for the extraction of plant-derived biomolecules from various feedstocks, with a notable focus on the extraction of molecules from citrus-based peels and seeds [34,61,90,91]. Resulting from this extraction method’s reliance on mechanical action, no external chemical input is required; hence, the resulting oil is 100% pure, retaining all its original properties [34,100]. Another commendable attribute of cold pressing and a likely reason for its adoption within industry is the health, safety, economic and environmental benefits resulting from the lack of chemical involvement [101]. Cold pressing has also been demonstrated to favour polyphenolic and antioxidant activity [101,102]. This may result from the lower operating temperatures (<40 °C), preventing the degradation of polyphenols and resulting in higher polyphenolic contents and antioxidant activity [103]. 

Cold pressing for the purpose of extraction may appear to be a simple approach to feedstock extraction; however, the pre-treatment methods necessary for some plant-derived biomolecules dictate the yield and extraction quality. Pre-treatment methods may include peeling, drying and the solvent or enzymatic treatment of raw materials [104], the specifics of which vary between species and target biomolecules [105]. Although several forms of presses exist, screw presses are the most common, offering a majority extraction in a single pass (typically leaving only 20% of the oil content within the output meal, while a second pass will leave 5–7% of the oil left in the resulting cake) [106]. An industrial-scale mechanical pressing plant has a typical throughput of 60–100 tons/day yet, while the throughput is high, the yield of mechanical pressing is typically relatively low. Consequently, a well-practiced strategy is the sequential use of solvent extraction with mechanical pressing (leaving only 1–2% of the oil content left in the final meal) [106,107,108].

### 2.4. Solvent Extraction

**Figure 5:**
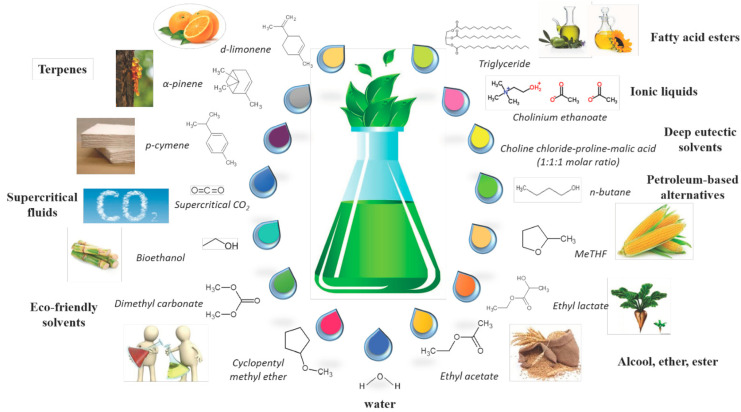
Examples of solvents used for extraction setups. Reproduced from [110] with permission (open access, Attribution 4.0 International (CC BY 4.0)).

Solvent extraction offers a selective approach to extracting bioactives, with the variation of the solvent controlling the yield/composition of the molecules isolated. Solvents with a polarity similar to a given solute often result in better extractions, and there is a trend towards the use of green solvents from renewable resources (examples of which are depicted in Figure 5) [110]. Various solvents, when used sequentially, may also be utilised to extract molecules with differing polarities [76,111,112]. Another benefit of solvent extractions is the opportunity for their implementation in extractions from fragile/delicate flowers, which may not be robust enough to tolerate the potentially degradative effects of heat intensive methods, such as steam distillation [60]. However, potential complications include the particle size of the raw materials, solvent to solid ratios, the extraction temperature and the duration [76].

For appropriate extraction, solvents require distillation or treatment prior to extraction in order to remove any impurities (e.g., stabilisers) that they contain, thereby minimising the prospects of complications. Moreover, solvents should be easily removable, inert, non-toxic and lacking flammability in order to enable simple processing [113] and minimise the potential for issues downstream (e.g., the presence of toxic residual solvents in the products) [114,115]. Consequently, techniques such as UAE are becoming ever more attractive [116,117]. 

A widely noted benefit of the greener methods, further to the reduction in solvent usage, is the emphasis on reducing the energy required to maintain the process as compared with organic solvent extraction methods [115,117,118,119]. Solvent extraction, particularly extraction through maceration, may require long extraction times and high solvent usage [76,119,120]; however, longer extraction times lead to higher costs [120].

### 2.5. Soxhlet Extraction

**Figure 6:**
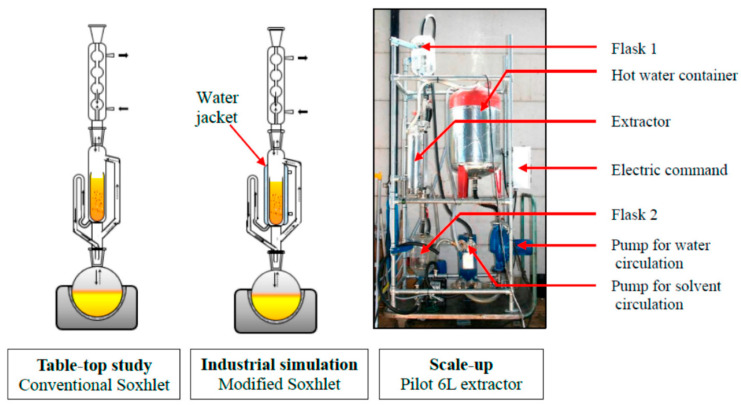
Schematic of Soxhlet extraction setups. Reproduced from [134] with permission (open access, Attribution 4.0 International (CC BY 4.0)).

Soxhlet extraction (depicted in Figure 6) is a method which was originally designed for lipid extraction from a solid matrix (e.g., leaves) [34] and is particularly useful for biological and environmental samples (e.g., soils, sediments, animal and plant material) [121,122]. Soxhlet extraction operates by using solvents such as dichloromethane, which may also be mixed with acetone or hexane, whilst non-polar solvents are typically not used [122]. While this technology is mature/established [123,124] and acts as a useful reference point for comparison against other extraction techniques [125,126], a disadvantage of this method is that the solvent utilised is the only parameter in the process allowing for selectivity, and subsequent concentration/purification may be required [127]. Potential drawbacks of Soxhlet extraction are its high energy/solvent consumption [127,128], long extraction times (with a typical minimum extraction time of approximately 8 h [122]) and reduced sample throughput [129]. Process optimisation for the extraction of crop oil from seed kernels can reduce extraction times to ca. 4.5 h [130], while in the case of pesticides, the extractions may be longer (6 to 24 h) [131]. Such differences in extraction times highlight the context dependence of each extraction method.

The prevalence of Soxhlet extraction is testament to the established advantages of the method. Soxhlet extraction is able to produce a higher yield of Eucalyptus oil from Eucalyptus leaves than HD (36.3% via Soxhlet extraction instead of 3.8% via HD). In addition to the higher yield, Soxhlet extraction enables the extraction of volatile components and high-molecular-weight molecules, as opposed to only volatiles in the use of HD [132]. Soxhlet extraction was also acknowledged to be better suited for the extraction of sterols, a type of lipid, from tobacco when compared with accelerated solvent extraction [133].

Similar to other extraction methods, given the parameters and context-driven nature of the investigation, the yields are variable. A study of the extraction of spent coffee grounds found that the oil yields obtained via Soxhlet extraction varied from 7 to 30% (dry weight); however, the authors noted that this may be due to variations in the feedstocks of spent coffee beans that were used [125]. The combination of innovative techniques, such as MAE/UAE, with Soxhlet extraction may prove capable of overcoming the challenges of using Soxhlet extraction alone [126].

### 2.6. Microwave-Assisted Extraction (MAE)

**Figure 7:**
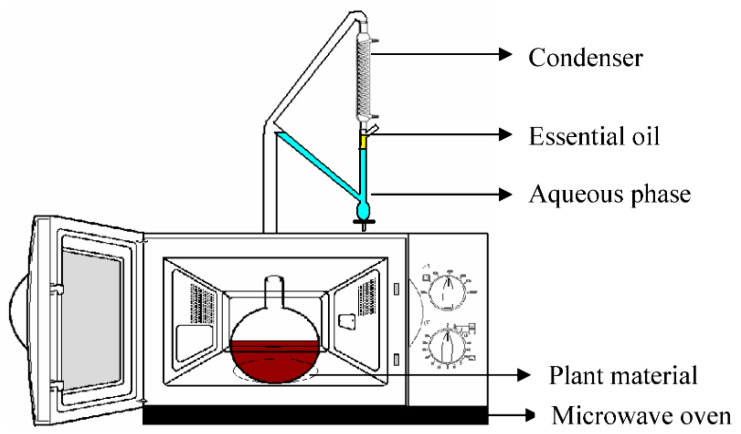
Schematic of the microwave-assisted extraction setup. Reproduced from [144] with permission (open access, Attribution 4.0 International (CC BY 4.0)).

MAE (in Figure 7) is used in conjunction with other forms of extraction techniques for the provision of auxiliary energy in order to increase energy savings, reduce solvent usage, decrease extraction times by up to 9 times and increase the product yield and quality, in accordance with the principles of ‘green’ chemistry [63,65]. MAE has a wide range of applications in extraction processes [135,136] and works in three stages. The energy provided through microwave irradiation increases the temperature and pressure in the extraction process, resulting in solute separation. The solute is released into the solvent from the sample matrix, and then the solvent becomes diffused across the sample matrix [137]. The efficiency of MAE, however, is still dependent on the solvent, sample and components under extraction, with the dielectric constant being an important parameter [135]. The dielectric constant, ε, is the ratio of the electric permeability of the material to the electric permeability of the free space [138], where the higher the constant is, the stronger the absorption will be. Thus, molecules reach the operational temperature quicker, and a closed vessel is preferred, allowing the solvent to be heated above its boiling point [139]. When the ε of the solvent is low, an open vessel is used, so that the sample components with relatively higher ε values will move into the surrounding cold solvent [140].

The rapid extraction of analytes coupled with the rapid heating of the sample–solvent mixture facilitates the extraction of thermally unstable compounds [135]. A comparison of the Soxhlet extraction method (6 h using hexanes) with MAE (20 s using hexanes) for the extraction of fresh peppermint oils showed higher yields obtained through the Soxhlet extraction; however, MAE produced a better quality extract, without the need for subsequent purification [64]. Interestingly, permutations of MAE, MAHD and SFME have led to reduced extraction times compared to HD in the case of citrus extractions, resulting in microwaves rupturing the biomass, without significant differences in the refractive index, specific gravity, visual appearance, colour or composition of extract. The significant reduction in CO_2_ emissions makes the MAHD and SFE methods appealing compared to HD, particularly on the large scale [141]. However, non-polar solvents are typically avoided in MAE due to their low absorption of microwave heating, although it is possible to add other chemicals in order to increase the microwave absorption or to carry out a pre-treatment with a polar solvent when non-polar solvents are a necessity [65]. There is potential for uneven heating and overheating due to variations in ε within the sample, reducing the extraction efficiency and leading to potential thermal degradation. There is also a requirement for downstream filtration steps in order to appropriately separate the residue from the liquid extract, and cooling times within the process must be considered for an optimal system to be designed [67,142]. These additional steps, as well as a clean-up step, may also lead to extract being lost through downstream procedures [143].

### 2.7. Ultrasound-Assisted Extraction (UAE)

**Figure 8:**
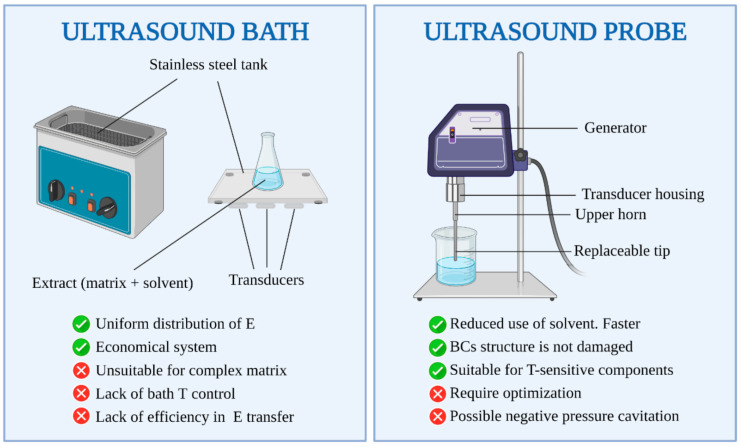
Schematics of ultrasound-assisted extraction setups. Reproduced from [155] with permission (open access, Attribution 4.0 International (CC BY 4.0)).

The ease with which ultrasound may be coupled with other extraction techniques and the ability to operate at room temperature minimizes the problems associated with the oxidation/decomposition of target products, successfully enabling the isolation of phenolic compounds, antioxidants and cyanine [145], which are important for producing cosmetics, nutraceutics and pharmaceutics [66]. UAE can be applied on both the microscale and macroscale to extract compounds derived from various forms of life [146], where the application of UAE is energy/solvent/time efficient, potentially offering high yields of delicate compounds [66,145,147]. The mechanical effects of ultrasound have wider implications than extraction alone, and by varying the power and frequencies, it can find applications both upstream and downstream, with a simple and inexpensive commercial integration [148] and optimisation [149]. A significant benefit of UAE is the simple equipment involved, as sonicating water-baths are widely available (as depicted in Figure 8). UAE is a less aggressive stimulus than MAE, which is therefore preferential for the extraction of unstable compounds (particularly in solvent extraction and maceration) [68]; however, UAE offers fewer modes of utilisation within extraction processes. UAE can be employed in both solvent extraction from the biomass and the evaporation process in order to enable solvent removal at lower temperatures, resulting in a greener process [150]. UAE may be faster/safer in the case of acid digestion, as it is possible to work at lower pressures/temperatures when using this method, and simpler procedures lead to lower contamination risks. However, the requirements of the particle size distribution of the feedstocks necessitates important pre-treatment steps, and the robustness of the probe surface may also be an issue [151]. In the case of extractions from grape seeds, UAE for 30 min gave similar results to Soxhlet extraction conducted for 6 h, with no significant differences in the fatty acid content. UAE used prior to maceration also allows for a higher polyphenol content [152]. The use of UAE has also proved beneficial in the extraction of anthraquinones, an aromatic compound, from *Heterophyllaea pustulata*, where the efficiency was >10 times more efficient with the use of UAE after 2 h compared to 16 h using the Soxhlet extraction method. Moreover, the efficiency and process time were improved further when UAE was coupled with subsequent MAE [153]. Comparing the phenolic compounds extracted from *Salvia officinalis* L. via UAE or water/solvent extraction, UAE resulted in a higher polyphenol extraction and lower solvent usage (under optimal UAE conditions, this equates to a 20% higher polyphenol extraction and up to a ~3-fold reduction in the process time) [154]. The aforementioned studies highlight the potential for utilising UAE for extractions, potentially coupled with MAE to further improve the extraction efficacy.

### 2.8. Subcritical Water Extraction (SWE)

**Figure 9:**
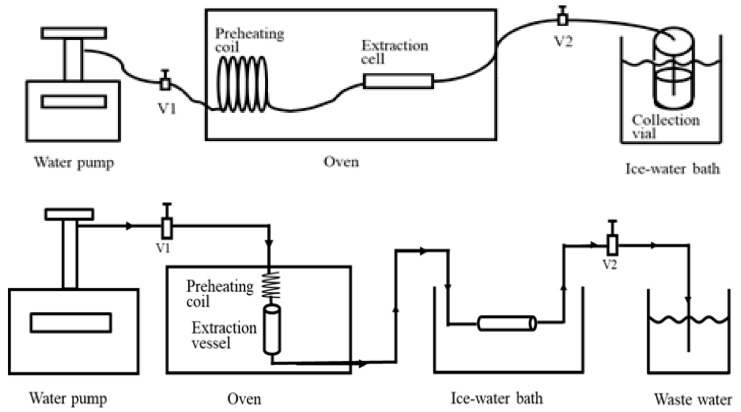
Subcritical water extraction system without solid trapping (top) and with solid trapping (bottom). Reproduced from [169] with permission (open access, Attribution 4.0 International (CC BY 4.0)).

SWE is a method that utilises only water in the extraction of compounds (including, but not limited to, antioxidants, carbohydrates, flavonoids, phenolics, proteins, etc.) from feedstocks (for example, *Chlorella vulgaris, Orostachys japonicus*, *Zataria multiflora*, etc.) [69,70,156]. The utilisation of subcritical water for extraction (as depicted in Figure 9) is flexible, offering users the ability to vary the temperature, hence altering the dielectric constant and, in turn, controlling the ability to solvate organic compounds with varying polarities [157]. Temperature is the most frequently varied parameter because of its effect on the dielectric constants, with room temperature enabling the solvation of polar compounds, and higher temperatures, in a subcritical state, enabling the solvation of low/intermediate-polarity molecules. Viscosity, surface tension and interactions with the matrix itself also play important roles in the SWE process [158]. Pressure within SWE plays a lesser role in the extraction process, as it does not have a dominant effect on the solvent characteristics, selectivity and efficiency, whereas temperature is the parameter with a dominant influence on the solvent characteristics through changing the dielectric constant [69,157,158]. SWE is an inexpensive and environmentally friendly mode of extraction (particularly due to its complete elimination of the use of potentially toxic organic) [159,160]. Moreover, it can be performed in either a dynamic or static state, or a state in between the two. However, a static state runs the risks of low recovery, low solubility or highly concentrated solutes, because water is not continually supplied, meaning that the extraction is limited, irrespective of the retention time, temperature or pressure. By comparison, a dynamic process may combat this issue, but the energy efficiency and volumes of the solvents used must still be considered [161]. Working at higher pressures facilitates extraction from samples, as the pressure forces water into the pores of the matrix, which is not typically subject to interactions [162]. However, a limitation of this method is that the high temperatures involved in this process may lead to the risk of thermal degradation of the molecules [163], potentially limiting the products to more thermally robust molecules and requiring the systematic optimisation in the case of a respective class of compounds in order to ensure the efficacy of the process [161,162]. A good complement to pilot experiments are solubility models and theoretical approximations, which can aid in predicting the solvent properties and/or the solubility of compounds within a solvent [164].

In a comparison between the continuous SWE of fennel with HD and solvent extraction, several benefits were identified. SWE facilitated a shorter extraction time of 50 min, compared to 4 h for HD and 24 h for solvent extraction. Costs were reduced via reduced energy requirements necessary to meet the SWE conditions, and, finally, the ability to alter the composition by changing the parameters of the temperature, flowrate and static extraction time was noted [165]. Similar benefits were identified for the extraction of *Lavandula stoechas* by SWE when compared with HD, but more important benefits were identified when this method was compared with UAE-solvent extraction. The aroma produced by SWE was also found to be more concentrated and powerful than that isolated by either HD or UAE-solvent extractions [166]. The former of these observations shows that SWE is competitive in relation with UAE and capable of producing value-added attributes, such as a stronger scent. Similarly, SWE, compared with the Soxhlet extraction (ethanol) and MAE-solvent (ethanol) extraction of Chaya, also showed promising results. Here, 10 min of SWE led to no significant differences in the global extraction yield compared to 6 h of Soxhlet extraction at similar temperatures, whereas 10 min of MAE produced approximately half the global extraction yield of Soxhlet (albeit that it yielded a product with greater antioxidant activity than that obtained by SWE or Soxhlet extraction) [167]. However, such considerations should be contemplated on a case by case basis, and some advantages and disadvantages of SWE have been discussed in a concise review [168].

### 2.9. Supercritical CO_2_ Extraction (scCO_2_)

**Figure 10:**
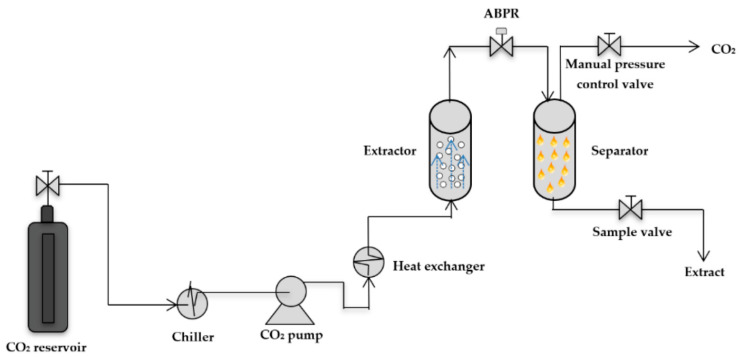
Schematic of the supercritical CO_2_ extraction setup. Reproduced from [185] with permission (open access, Attribution 4.0 International (CC BY 4.0)).

Supercritical fluid extraction has been employed to extract valuable components from numerous feedstocks [71,170,171,172]. The broad applicability of scCO_2_ (depicted in Figure 10) can be attributed to the fact that the selectivity and solvation characteristics may be optimised by the manipulation of the temperature and pressure [173]. Supercritical fluids can facilitate much higher diffusion coefficients of lipids and waxes than other liquids, allowing for quicker extractions. The lack of surface tension within supercritical fluids, coupled with lower viscosities, enables a greater penetration into the pores of a matrix compared with other liquids [174]. Extractions using scCO_2_ simplify the downstream processing and handling, particularly because CO_2_ is inflammable, relatively inexpensive and, when handled carefully, non-toxic. The separation of molecules from plant matter is also relatively easy as, once adjusted to ambient conditions, CO_2_ readily evaporates, requiring less downstream processing, and the low critical temperatures required for CO_2_ reduce the potential for problems related to the thermal degradation of the molecules [175]. Importantly, scCO_2_ is regarded as a green technology [174,176]. While the equipment necessary for scCO_2_ processing is not cheap, it is a process in which the oxidation of the target molecules is minimal [177]. Due to the fact that scCO_2_ is a non-polar solvent, it is optimal for the extraction of non-polar and weakly polar compounds [178,179]. However, when coupled with polar organic solvents, such as methanol or ethanol, as co-solvents, the extraction efficiency of the polar compounds may be increased [179,180] (and the integration of (bio)ethanol is considered to be environmentally benign and relatively safe for human health). Interestingly, the use of scCO_2_ may facilitate the extraction of a wider portfolio of compounds, whereas conventional methods such as HD and solvent extraction are optimal for compounds that are volatile or high in molecular weight [181].

A study of the isolation of tocochromanols from cereals demonstrated that scCO_2_ was comparable to Soxhlet extraction in terms of the yield (~85%), but the benefits of scCO_2_ processing included its low solvent usage and ease of analysis [182]. It was reported that scCO_2_ gave a slightly lower yield of isoflavones from soybeans than conventional extraction using 80% ethanol solvent, but this was counterbalanced by improvements in the processing due to the use of scCO_2_ (i.e., the lack of use of organic solvents) and its ease of clean-up and analysis [183]. Moreover, it was noted that the integration of ethanol into the scCO_2_ extraction process (92.5% CO_2_ and 7.5% ethanol) improved the yield of isoflavones [183]. The investigation of the extraction of secondary metabolites from *Syzygium campanulatum* using scCO_2_ with added ethanol resulted in a higher recovery (of 25–85%) compared with conventional solvent extraction, with values ranging from 0.9–66% [184], highlighting the potential benefit of adding an organic solvent to the extraction process (some advantages and disadvantages of scCO_2_ extraction are discussed in a concise review [168]).

## 3. Analytical Methods

**Figure 11:**
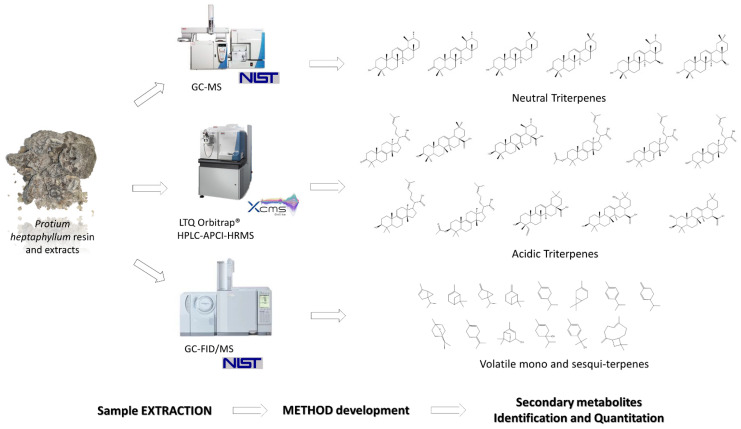
Examples of natural products analysed by variants of GC-MS and HPLC-MS. Reproduced from [191] with permission (open access, Attribution 4.0 International (CC BY 4.0)).

A variety of analytical techniques can be utilised when extracting high-value chemicals from plants. Chromatographic separation enables the isolation of individual compounds from mixed feedstocks and their subsequent analysis via crystallography, spectrometry, spectroscopy, etc. [186]. Modern approaches to this technique involve combinations of methods for the separation and analysis, where a chromatographic technique is coupled with spectrometry, giving rise to methods such as gas chromatography–mass spectrometry (GC-MS) or high-performance liquid chromatography–mass spectrometry (HPLC-MS) [187,188], both of which are suitable for low-molecular-weight compounds [189]. Gel permeation chromatography (GPC)–matrix-assisted laser desorption ionization (MALDI) mass spectrometry, on the other hand, is more suitable for high-molecular-weight compounds. GC-MS and HPLC-MS can produce valuable results, even when the yield of the extraction process may be low (potentially too low for other methods that require significant amounts of the sample, e.g., nuclear magnetic resonance spectroscopy) [190]. Examples of natural products analysed by variants of GC-MS and HPLC-MS are depicted in Figure 11 [191].

Antioxidant potential and oxidative stability are used to examine the bioactivity of the compounds extracted from feedstocks. Oxidative processes have been noted to reduce the quality and pharmacological activity of plant-derived chemicals [192] and, since oxidative stability is synonymous with the shelf-life of the extract [193,194], it is a useful determinant for predicting its longevity. Factors influencing oxidative stability include the composition of the extract itself and its respective antioxidant capabilities, as well as the pro-oxidant effects of the environment on the extract during processing and storage [195], which include heat, light, oxygen availability and the presence of trace metals [196,197,198]. Antioxidant potential (which is associated with oxidative stability [195]) correlates with the shelf-life of a product and is important in the food industry for maintaining nutritional quality [199,200].

### 3.1. Gas Chromatography Mass Spectrometry (GC-MS)

GC-MS is an analytical technique routinely used to analyse volatile, thermally stable compounds [201,202]. Certain species may be too polar or large to be sufficiently volatile in order to pass from the liquid phase to the mobile gas phase prior to analysis via MS [203], and this limitation can be overcome by derivatization (i.e., chemical modification, ion-pairing techniques, photochemistry, electrochemistry, complexation and metal chelation) [204], which alters the physical or chemical characteristics to improve the sensitivity or selectivity within the analysis [205]. Derivatization should create sufficiently volatile and thermally stable compounds, which increases the sensitivity, selectivity or specificity within the analysis [206,207], thereby increasing the scope of the analytes that GC-MS may be used for [203,208,209]. GC-MS offers a high resolution and reproducibility within chromatographic analysis [210,211]. The complexity of the sample preparation limits the size and type of the molecule under analysis [209], and whilst this can be mitigated to a certain extent by derivatisation, derivatization can be complicated by the selectivity of functional group modification and other issues [212].

The use of GC-MS in plant extract analysis is popular because of its ability to identify a plethora of bioactive phytochemicals from a given feedstock employing large spectral libraries [213]. The analysis of an ethanolic extract of *Evolvulus alsinoides* (L.) identified its potential chemo-preventive, anti-cancer, anti-microbial, antioxidant and anti-diabetic activity due to the presence of secondary metabolites in the extract [214]. The phytochemical analysis of the ethyl acetate and methanolic extract of *Amomum nilgiricum* via GC-MS identified 25 phytochemicals with potential antibacterial, antifungal, antiviral, antioxidant and antidiabetic properties [215]. The GC-MS analysis of an ethanolic leaf extract of *Phyllowdium pulchellum* L. identified 10 phytochemical compounds with anti-inflammatory, antioxidant, anti-microbial, anti-malaria, anti-fungal, cytotoxic and hypoglycaemic activity [216]. Numerous studies have also presented similar findings in the case of plant extracts whilst using GC-MS as a tool of analysis [217,218,219,220], which can be cross-referenced against a database for compound identification and biological property analysis/prediction [217]. The aforementioned studies highlight the power of GC-MS as an analytical tool for identifying commercially viable, bioactive and therapeutic molecules from plant extracts. For the avid reader, we recommend the state-of-the-art of the literature on GC-MS [221,222,223].

### 3.2. High-Performance Liquid Chromatography Mass Spectrometry (HPLC-MS)

HPLC-MS is a potent technique for separation via HPLC followed by mass analysis via MS [224,225]. The benefit of HPLC-MS, compared to GC-MS, is the fact that the analytes do not need to be volatile in order to be analysed [226], enabling the broad applicability of HPLC-MS for the separation, identification and quantification of small molecules with a high sensitivity/selectivity in trace multicomponent and complex mixture analysis [227,228]. Similar to GC-MS, HPLC-MS may employ derivatization in order to improve the detection characteristics, compound stabilization and binding to HPLC columns, thus facilitating easy compound separation [229]. LC-MS offers versatility in the mode of LC operation with reverse-phase liquid chromatography (RPLC), normal-phase liquid chromatography (NPLC), hydrophilic interaction liquid chromatography (HILC), ion exchange chromatography (IELC) and size exclusion chromatography (SELC) [230,231]. RPLC is popular in the analysis of metabolomics [232,233], whilst HILC is used for polar molecules to increase the range of the compounds that may be investigated by LC [232,234], with IELC expanding the scope of LC-MS even further for the analysis of highly polar molecules [232,235]. The versatility of LC-MS facilitates the analysis of a wide range of molecules and, importantly, mixed-mode chromatography may also be utilised, which combines two or more of the aforementioned techniques in order to offer superior separations than those of the individual methods alone [236]. This underpins the popularity of HPLC-MS as a tool for the analysis of a plethora of analyte families. For the avid reader, we recommend the literature on the various state-of-the-art chromatographic techniques [237] that are coupled with MS: LC [238], HILC-MS [239], HPLC-MS [240], IELC-MS [241,242] and SELC-MS [243,244].

## 4. Conclusions

Chemicals produced by plants (e.g., alkaloids, flavonoids, phenolic acids, saponins and terpenes) have a variety of high-value applications in industry. Extraction techniques used to isolate mixtures containing these compounds have been developed, which employ complex combinations of chemical/engineering methods used to extract crude oils. The desire to find more sustainable/circular solutions has motivated the development of innovative industrially scalable extraction methods (e.g., the provision of auxiliary energy via microwaves and ultrasound, which enables quicker/greener processing via reductions in the organic solvent requirements, energy loads and waste production, thereby increasing the efficiency). Subcritical water and supercritical CO_2_ have been shown to be effective greener solvents, with reduced heating requirements and waste. Such extraction techniques are typically coupled with analytical techniques, such as GC-MS and HPLC-MS, to identify and potentially quantify the valuable compounds. While there are context-specific variations in the yields of high-value chemicals from plants (including the extraction methodology and feedstock), the application of the techniques outlined herein offers the potential for significant economic, environmental and societal impacts in the future.

## Figures and Tables

**Table 1:** Essential oils for common medical problems. Adapted from the literature [32] (open access, CC BY-NC-ND 4.0).

Condition(s)	Essential Oils
Anxiety, agitation, stress, challenging behaviours	*Angelica archangelica rad.* (angelica), *Cistus ladaniferus* (labdanum), *Citrus aurantium var. amara fol.* (petitgrain bigarade), *Citrus aurantium var. amara per.* (orange bigarade), *Citrus bergamia* (bergamot), *Citrus sinensis* (sweet orange), *Cymbopogon martinii* (palmarosa), *Eucalyptus staigeriana* (lemon-scented ironbark), *Lavandula angustifolia* (lavender), *Litsea cubeba* (may chang), *Ocimum basilicum* (basil), *Origanum majorana* (sweet marjoram), *Pelargonium graveolens* (geranium), *Pogostemon patchouli* (patchouli), *Valeriana officinalis* (valerian)
End-of-life agitation	*Lavandula angustifolia* (lavender), *Santalum album* (sandalwood), *Boswellia carteri* (frankincense)
Fatigue	*Angelica archangelica rad.* (angelica) (nervous), *Cistus ladaniferus* (labdanum) (chronic), *Citrus aurantium var. amara* (neroli bigarade), *Citrus paradisi* (grapefruit) (exhaustion), *Coriandrum sativum* (coriander) (including mental), *Cymbopogon nardus* (citronella), *Eucalyptus radiata* (black peppermint) (chronic), *Eucalyptus smithii* (gully gum), *Juniperus communis ram.* (juniper twig), *Mentha spicata* (spearmint) (mental), *Pelargonium graveolens* (geranium) (nervous), *Pinus sylvestris* (Scots pine), *Rosmarinus officinalis ct. cineole, ct. camphor, ct. verbenone* (rosemary), *Salvia sclarea* (clary) (nervous), *Zingiber officinale* (ginger)
Insomnia	*Angelica archangelica rad.* (angelica), *Cananga odorata* (ylang ylang), *Chamaemelum nobile* (Roman chamomile), *Citrus aurantium var. amara* (neroli bigarade), *Cistus ladaniferus* (labdanum), *Citrus bergamia* (bergamot), *C. limon* (lemon), *Citrus reticulata* (mandarin), *Citrus sinensis* (sweet orange), *Cuminum cyminum* (cumin), *Juniperus communis fruct.* (juniper berry), *Lavandula angustifolia* (lavender), *Litsea cubeba* (may chang), *Melissa officinalis* (lemon balm), *Myrtus communis* (myrtle), *Ocimum basilicum* (basil) (nervous), *Origanum majorana* (sweet marjoram), *Ravensara aromatica* (ravensara), *Thymus vulgaris ct. geraniol, ct. linalool* (sweet thyme), *Valeriana officinalis* (valerian)
Mental exhaustion, burnout	*M. piperita* (peppermint), *Ocimum basilicum* (basil), *Helichrysum angustifolium* (everlasting)
Memory loss	*Litsea cubeba* (may chang), *M. piperita* (peppermint), *Rosmarinus officinalis ct. cineole* (rosemary)
Pain management	*Eucalyptus smithii* (gully gum), *Lavandula angustifolia* (lavender), *Matricaria recutita* (German chamomile), *Leptospermum scoparium* (manuka), *Origanum majorana* (sweet marjoram), *Pinus mugo var. pumilio* (dwarf pine), *Rosmarinus officinalis ct. camphor* (rosemary), *Zingiber officinale* (ginger)

## Data Availability

Not applicable.
